# Soybean Protein and Oil Variants Identified through a Forward Genetic Screen for Seed Composition

**DOI:** 10.3390/plants11212966

**Published:** 2022-11-03

**Authors:** Karen Hudson

**Affiliations:** USDA-ARS Crop Production and Pest Control Research Unit, 915 West State Street, West Lafayette, IN 47907, USA; karen.hudson@usda.gov; Tel.: +1-765-494-8057

**Keywords:** soybean protein:oil, soybean protein content, soybean oil content

## Abstract

Mutagenesis remains an important tool in soybean biology. In classical plant mutation breeding, mutagenesis has been a trusted approach for decades, creating stable non-transgenic variation, and many mutations have been incorporated into germplasm for several crops, especially to introduce favorable seed composition traits. We performed a genetic screen for aberrant oil or protein composition of soybean seeds, and as a result isolated over 100 mutant lines for seed composition phenotypes, with particular interest in high protein or high oil phenotypes. These lines were followed for multiple seasons and generations to select the most stable traits for further characterization. Through backcrossing and outcrossing experiments, we determined that a subset of the lines showed recessive inheritance, while others showed a dominant inheritance pattern that suggests the involvement of multiple loci and genetic mechanisms. These lines can be used as a resource for future studies of the genetic control of seed protein and oil content in soybean.

## 1. Introduction

Although CRISPR and gene editing technologies show great promise in soybean composition engineering [1,2,3] stable soybean transformation remains a significant challenge for most laboratories, and this technology has not yet reached the level of low cost and rapid throughput where it can be applied to broad hypothesis testing in the public sector. Mutagenesis followed by forward genetic screening still has the advantage in soybean that only mutations that are phenotypically significant even in the presence of often redundant, highly similar homeologous genes are detected. For seed composition characteristics, traits created via mutagenesis have the added benefit of being categorized for regulatory and labelling purposes as non-genetically modified, which can accelerate study and broader adoption. Before the genomic age, forward genetics was a time- and resource intensive process that took many years, particularly in soybean, but today soybean genetic research can take advantage of many genomics-enabled sequencing technologies to accelerate the process of map-based cloning, including genotyping by sequencing and other high throughput dense marker approaches, and whole-genome resequencing. As a tool for the discovery of gene function, chemical mutagenesis can still provide validated targets to affect gene function in complex genomes like soybean, and several groups are currently exploiting this approach [4,5,6].

### Seed Protein and Oil Content Are Complex and Correlated Traits

Soybean is an important source of protein and oil for food and animal feed. In general, commodity soybeans are 40% protein and 20% oil by dry mass, the remainder being composed of carbohydrate compounds and minerals. An ideal composition for a commodity soybean would maximize levels of protein and oil, while reducing carbohydrates, in particular the difficult-to-digest oligosaccharides. More natural genetic variation exists for protein content than for oil in the soybean germplasm, although oil quality has been improved by both genetic and biotechnological approaches, and the oil fraction of soybeans remains important as a food oil and a source fo renewable energy as biodiesel [7]. A minimum of 41.5% protein on a dry weight basis is necessary for soybean to produce meal sufficiently nutritious for use in swine and poultry feed, and the demand for soybean meal is a primary driver for the value of the soybean crop. Seed protein and oil content have been demonstrated in many studies to have an inverse relationship, making it difficult to improve protein levels while maintaining satisfactory levels of seed oil [8]. Seed protein content is also often negatively correlated with overall yield, a factor that has further inhibited high-protein germplasm development [9,10,11]. Primarily for these reasons, breeding and transgenic approaches have thus far had limited impact in the creation of new soybean varieties with increased protein content [12]. Increasingly, soybean processors and meal and feed formulators are interested in soybean with higher protein levels, which underscores the need to understand the interactions among genes that determine seed protein content.

While 95% of U.S. soybeans are destined for animal feeding applications, the established production infrastructure makes soybean poised to capture the current increased consumer interest in plant-based protein for health and environmental concerns. As this industry evolves, seed composition improvement drives value for both commodity soybeans and high-value food crop opportunities. While much is known regarding genetic pathways involved in regulating levels of the oil and carbohydrate constituents of soybean seed, which are generally controlled by linear biosynthetic pathways, the genetic control of overall levels of seed protein and oil is poorly understood at the molecular level.

Soybean seed protein content has been a subject of study for decades. Seed protein levels exhibit diversity across germplasm accessions, and hundreds of quantitative trait loci (QTL) have been identified that influence seed protein. It is likely that many of these are overlapping regions that demarcate the location of several important genes, however QTL and genome wide association (GWAS) studies on seed protein are complicated by the sensitivity of seed composition traits to the growing environment [13]. Only 16 QTL for seed protein have been designated “Confirmed QTL” by the Soybean Genetics Committee on the basis of having been well-mapped across numerous studies and populations. Two of these have been recently identified genetically. On chromosome 20, an insertion/deletion in a CCT-domain protein is highly correlated to protein levels in populations segregating for this variation, and RNAi knockout of the mis-spliced form results in elevated protein levels [14]. On Chromosome 15, a *SWEET* gene encoding a sugar transporter underlies a major QTL for seed protein, oil, and seed size associated with soybean domestication. When knocked out, *sweet10a* mutants have reduced levels of protein [15,16]. Interestingly, both genes are expressed in the seed coat during development, underscoring the importance of this tissue in the transport of nutrients into the seed. However, few QTL have been used to generate successful high-protein soybean varieties through marker-assisted breeding, largely because the loci with larger effect sizes that raise protein level tend to carry a yield penalty [9,11]. Model system research has provided a number of targets for seed composition engineering, however these have yet to be associated with the natural QTL and implies that there are further loci that could be uncovered through genetic studies [17]. This underscores our need to further understand the complex genetic and molecular basis of composition in soybean seeds, and how it is affected by environmental conditions. Mutagenesis approaches are useful tools to generate new variation, and may illuminate genetic mechanisms with changes distinct from those available in wild populations.

In this study, we have utilized a forward genetic approach to create new variation and new sources for high protein (or high oil) soybeans and to further our understanding of the environmental and genetic control of resource allocation within the seed. By following the seed composition over multiple seasons and through genetic test crosses, we obtain preliminary characterization, an estimation of the reproducibility of the trait, and prioritize the mutants for further study.

## 2. Results

### 2.1. Identification of Mutants

**Figure 1:**
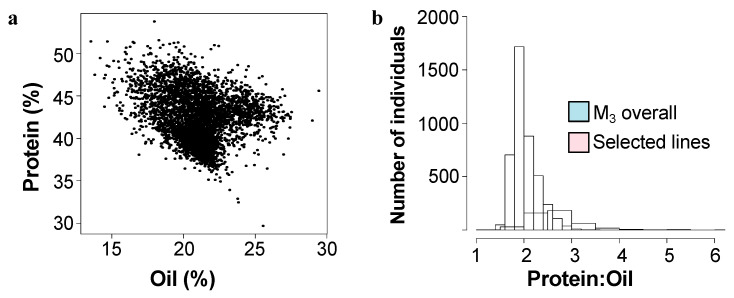
Mature seed protein and oil content in the mutant population. (**a**) Total protein and oil content in mature seeds as determined by NIR as % dry weight basis for 4300 M_3_ lines of the mutant population. (**b**) Distribution of mature seed protein:oil ratio for all mutant lines (blue) and the selected mutant lines (pink) over five seasons.

A population of over 8000 inbred Williams-82 soybean was treated with N-methyl nitrosourea (NMU) to induce single nucleotide polymorphsims (SNPs). The intended purpose of this population was for a TILLinG (Targeting Induced Local Lesions in Genomes) approach for reverse genetics, and it has provided new alleles for the modification of carbohydrates in soybean seeds [18,19]. However, it has also proven fruitful as a source of new composition alleles using a forward genetics approach by screening for composition phenotypes [5]. Over the course of five field seasons, 4300 M_3_ lines (each line representing the offspring of one M_2_ mutant individual) were screened by NIR (Near-InfraRed Spectroscopy) for overall protein and oil levels in seeds (Figure 1a).

**Figure 2:**
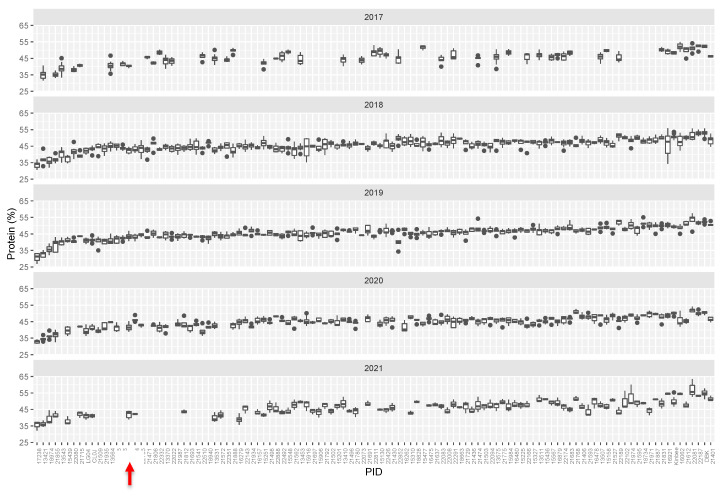
Tukey box plots of average seed protein content (%, dry weight basis). Seed protein content in M3 lines over five growing seasons. Lines are plotted left to right in order based on increasing median protein level over 5 years. Abbreviations: W82—Williams-82, LG04—LG04-6000, CL0J: CL0J-173-6-8, DBK: Danbaekkong. Red arrow indicates the Williams-82 (wild type) content.

We initially identified over 125 lines that varied from the reference genotype (Williams-82) by ~>10% in protein and/or oil content. Most commonly, mutants were elevated in protein levels and were reduced in levels of total oil, however seven mutant lines had elevated levels of oil and reduced levels of protein. Compared to the initial full mutant population, ratio of protein to oil in the selected mutants tended to be higher, driven by several outlying lines with low levels of seed oil (Figure 1b). Mutant rows which had low seed set due to reduced fertility were eliminated, as protein levels are inversely correlated with seed set. Promising lines, which showed statistically significant differences from the Williams-82 wild-type were followed for up to five subsequent growing seasons. (Figure 2, Supplemental Table S1). Many of the lines demonstrated statistically significant and reproducible effects on protein and/or oil content over multiple years. Many of the mutants demonstrated agronomically significant increases in protein levels (up to 50% protein, similar to known high protein lines such as Danbaekkong (PI 619083) or Kinbee (PI 417027). LG04-6000 (PI 664025, [20]) and CL0J-173-6-8 [21] are locally adapted commodity-type high-yielding soybean, and were characterized in the SoyNAM project, thus have publicly available dense genome marker data [22].

### 2.2. Genetic Classification of Mutants

To further characterize the most promising mutant lines, mutants were backcrossed to Williams-82 (with the mutant as the male parent) and outcrossed to another parent for genetic mapping (with the mutant as the female parent). Small populations (25–60) of F_3_ seed from an individual cross were phenotyped by NIR (Table 1). As overall variation for these quantitative traits is relatively subtle, it was important to determine if the phenotypes can be followed in a segregating population as the result of a single locus. In many cases it was possible to determine if each locus was dominant or recessive. In general, visual inspection of the protein and oil levels in F_3_ seed bulks was used to assess the inheritance pattern. Statistical methods were applied to attempt to validate segregation in the population, although these have limited effectiveness with small populations (Table 1).

**Figure 3:**
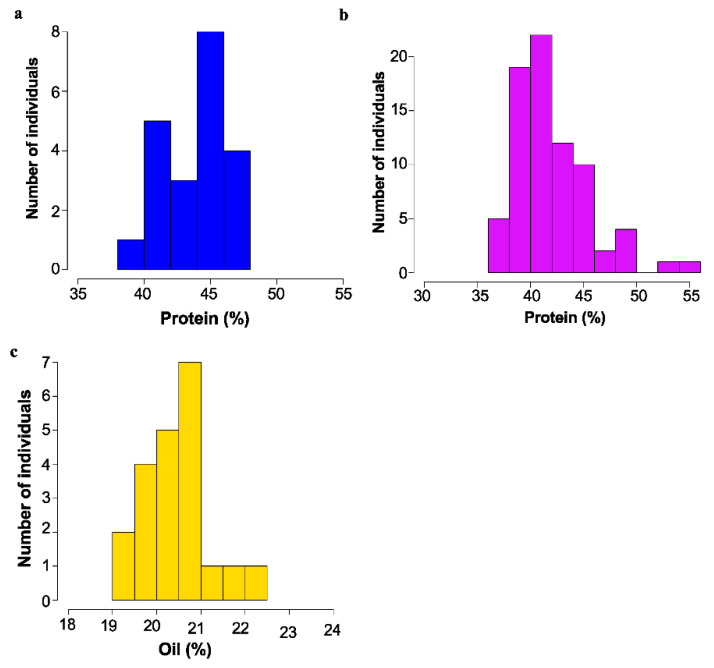
Mature seed protein or Oil content as determined by NIR as % dry weight basis. for mutant backcross populations. (**a**) Segregation of protein content in 16475 × Williams-82 F_3_; bulk seed samples. (**b**) Segregation of protein content in 18734 × Williams 82 F_3_ bulk seed samples. (**c**) Segregation of oil content in 17238 × Williams 82 F_3_ bulk seed samples.

Of 44 lines crossed, it was determined that 14 of the lines showed a dominant, high protein phenotype, and 17 lines showed a recessive high protein phenotype. Two were classed as recessive high oil, and one was recessive low oil. For the remainder of the cases, the cross was not informative. Segregation of protein and oil levels for several example populations are shown in Figure 3.

## 3. Discussion

A significant challenge for identifying new protein and oil mutants in soybean is the number of individual plants and volume of seed that must be screened. Ideally, for testing maintenance of the genetic trait over multiple seasons and segregation within populations, large populations are superior, however in a screen a balance must be achieved between the time spent propagating and characterizing individuals and a wide survey of the mutant population and preliminary characterization of numerous lines to achieve the best return of potential new loci. NIR-based methods have the strength of being fast, non-destructive, and with immediate relevance to how protein is measured in industrial settings, however it is important to validate this with chemical or biochemical approaches as the assumptions of NIR calibrations to infer protein amounts may not hold true in developmental mutants, for example those that affect the seed coat [23,24].

A key difference between the lines that we have identified from the mutant population and previous high-protein lines that have been the focus of composition research is that prior work has focused on a limited number of major protein QTL from diverse genotypes [9,25]. Soybean is limited in diversity and has undergone genetic bottlenecks during domestication [26]. The mutagenesis approach creates new diversity that did not previously exist in soybean. Combining these two approaches, a simple point mutation that is allelic with a more complex germplasm allele can help identify the causative gene underlying a QTL isolated from a germplasm source. The genetic differences between germplasm accessions are often a result of large genetic deletions, duplications or complex genomic rearrangements, which makes them hard to interpret in terms of gene function. Mutants from this chemically mutagenized population are expected to be the result of a single-base point mutation in the Williams-82 genetic background. (For all the fatty acid mutations previously characterized from this population, we have found this to be the case [27].) Thus, identification of the molecular nature of the lesion in these mutant lines should be more straightforward following genetic mapping.

Interestingly, we have observed both recessive and dominant phenotypes for the control of overall protein levels, which implies that multiple loci have been identified, and as SNP polymorphisms frequently create loss-of-function alleles this suggests multiple mechanisms can affect mature seed composition. In addition to using a mutation breeding approach to increase protein, mutants with reduced protein levels can confer valuable information about resource allocation during seed development. It is likely that by identifying genes involved in the control of protein levels we will gain understanding on how the balance of storage compounds in seeds is regulated and find new ways to improve composition, for example by changing gene activity in an opposite direction using transgenic or targeted mutation approaches.

## 4. Materials and Methods

Williams-82 soybean were mutagenized with n-nitroso-n-methylurea (NMU) as described previously [28]. M_2_ soybeans were planted in 1.8m plots containing up to 25 plants at the Agronomy Center for Research and Education field in West Lafayette, Indiana (40.0700° N, 86.9918° W). Over the five growing seasons (1 June–1 September) for each year at the experimental location, the average daily temperature ranged from 27.4 °C in 2017 to 28.8 °C in 2021. Rainfall during the growing season averaged 32.4 cm, and ranged from 19.7 cm in 2019 to 46.5 cm in 2017 (Midwestern Regional Climate Center, West Lafayette, IN, USA, https://mrcc.purdue.edu/CLIMATE/welcome.jsp, accessed on 1 November 2022). Seed total protein and oil from M_2:3_ were measured on bulks of 15 seed using the mirror cup of a Perten DA 7250 NIR analyzer (Perten, Springfield, IL, USA). M_3_ seeds were planted in the field and harvested as individual plants, and protein and oil was measured in M_4_ seeds to determine if the mutation was segregating (suggesting a heterozygous state in the M_2_) or uniform (indicating a homozygous line determined by overall consistency and small standard deviation among individuals). M_2_ families were assigned and followed with a unique, five-digit PID (Plant ID) number. In Supplemental Table S1, protein or oil is expressed as an average of three or more individuals, and statistical significance was determined by two-tailed, type II *t*-test with Williams-82 wild type individuals from the same year, which was the method that determined if a particular M_2_ line would be planted in future seasons. For lines where the trait was assessed to be segregating, plants were self pollinated and individuals from further generations were tested until the selfed progeny showed consistent protein or oil levels (at which point it was presumed that the gene involved was in the homozygous state). Presumed homozygous mutants were backcrossed to Williams-82 as the male parent (such that in the F_3_ seeds, deviation from wild-type protein or oil levels indicated a successful backcross, as no flower color, pubescence, or other physical characteristics can be used to differentiate the mutants from the non-mutagenized parent to determine if the cross was successful). Statistical analysis of M_2_ families and F_2_ populations, including the Shapiro–Wilks test for normality [29] were performed in R and Microsoft Excel, and plots were generated in R.

## Figures and Tables

**Table 1:** Inheritance pattern of alleles for high protein from selected mutants.

Backcross PID	Phenotype	No. of Plants	SW	*p*
13421	inconclusive	39	0.914	0.006
13507	D high protein	50	0.975	0.380
13531	r high protein	48	0.974	0.352
14015	r high protein	55	0.939	0.008
15130	r high protein	56	0.988	0.840
15158	D high protein	56	0.978	0.405
15201	inconclusive	19	0.983	0.972
15251	D high protein	85	0.936	<0.001
15310	r high protein	107	0.992	0.763
15439	r high oil	70	0.986	0.607
15477	r high protein	35	0.978	0.704
15548	r high protein	40	0.930	0.017
15906	r high protein	22	0.890	0.019
15916	r high protein	100	0.993	0.893
16062	D high protein	96	0.971	0.032
16157	inconclusive	37	0.953	0.119
16262	r high protein	38	0.980	0.734
16279	D high protein	54	0.964	0.108
16475	D high protein	56	0.950	0.021
16480	r high protein	38	0.951	0.094
16879	D high protein	42	0.920	0.006
16921	D high protein	48	0.950	0.040
17238	r high oil	44	0.872	<0.001
18663	r high protein	78	0.969	0.052
18734	r high protein	76	0.907	<0.001
18940	inconclusive	32	0.974	0.626
18974	r high protein	39	0.967	0.295
21401	D high protein	49	0.972	0.288
21424	D high protein	54	0.975	0.325
21502	inconclusive	41	0.980	0.687
21715	r low oil	51	0.964	0.125
21768	inconclusive	42	0.991	0.982
21775	r high protein	31	0.963	0.357
21831	D high protein	58	0.987	0.793
21855	r high protein	48	0.959	0.091
21887	D high protein	57	0.982	0.552
22080	Inconclusive	41	0.950	0.071
22081	Inconclusive	30	0.975	0.688
22102	r high protein	38	0.924	0.013
22143	D high protein	40	0.939	0.031
22187	Inconclusive	56	0.988	0.841
22189	Inconclusive	53	0.970	0.199
22426	D high protein	31	0.953	0.195

PID—Plant ID Number, SW—Shapiro–Wilks statistic in test for normality, *p*—*p*-value. D—dominant, r—recessive.

## Data Availability

Not applicable.

## Supplementary Materials

The following supporting information can be downloaded at: https://www.mdpi.com/article/10.3390/plants11212966/s1, Table S1: Protein and oil content of selected mutants over five growing seasons.
