# Supplementary material for: Soybean Protein and Oil Variants Identified through a Forward Genetic Screen for Seed Composition
Source: Plants (Basel). 2022 Nov 3;11(21):2966. doi: 10.3390/plants11212966 (PMC9656176; doi:10.3390/plants11212966)
Supplement: Supplementary file 1 [file plants-11-02966-s001.zip › plants-1974793-supplementary.pdf]

Supplemental Table S1. Protein and oil content of selected mutants over five growing seasons

	2017			2018			2019			2020			2021		
	Protein	Oil	n	Protein	Oil	n	Protein	Oil	n	Protein	Oil	n	Protein	Oil	n
13370	43.49 ± 3.0	18.75 ± 1.5*	8	44.58 ± 2	19.93 ± 1	3	44.21 ± 2.3	18.66 ± 0.8	5	41.42 ± 2.1	18.55 ± 0.2	5	nd	nd	
13410	44.04 ± 2.1**	17.58 ± 1.3*	19	45.82 ± 1.1**	18.25 ± 0.8**	7	47.39 ± 0.7**	17.13 ± 0.5**	5	46.26 ± 1.1**	14.11 ± 1.8**	5	48.58 ± 3.8*	16.83 ± 2.3*	3
13421	35.19 ± 2.7*	20.96 ± 1.0	17	37.01 ± 3**	21.28 ± 1.9	8	32.74 ± 2**	22.55 ± 0.8**	5	34.62 ± 1.4**	19.3 ± 0.6	5	36.16 ± 2*	22.14 ± 0.2	3
13453	44.42 ± 3.0**	14.92 ± 1.1*	9	43.91 ± 2.1	16.29 ± 2**	8	43.83 ± 3.1	16.34 ± 3.5**	5	45.57 ± 0.8*	11.86 ± 1.3**	5	49.58 ± 0.8*	13.93 ± 1.2**	3
13507	45.85 ± 2.3**	17.64 ± 1.3*	8	48.91 ± 1.8**	16.63 ± 1.3**	8	48.66 ± 1.8**	15.75 ± 0.8**	5	47.81 ± 2.6**	15.38 ± 1.3**	5	47.73 ± 1.9*	17.68 ± 0.4*	3
13511	46.91 ± 1.7**	15.34 ± 2.2**	16	46.67 ± 2**	12.17 ± 2.4**	5	46.91 ± 2.9**	16.26 ± 1.8**	5	45.24 ± 1.5*	11.07 ± 2.5**	5	51.27 ± 2.1**	10.44 ± 1.1**	3
13531	44.88 ± 2.6*	17.69 ± 1.7*	12	42.49 ± 1.6	18.4 ± 1**	8	44.98 ± 1.6	17.01 ± 1.3**	5	42.63 ± 2.2	17.02 ± 1.5*	3	39.97 ± 3	21.51 ± 1.1	3
13543	39.02 ± 2.5	19.23 ± 2	24	40.41 ± 3.1	17.61 ± 1.6**	8	40.06 ± 2**	17.53 ± 0.8**	5	nd	nd		nd	nd	
13564	40.74 ± 2.7	20.03 ± 1	13	45.26 ± 2*	18.96 ± 1.4*	8	41.2 ± 2.5*	17.46 ± 0.8**	5	44.73 ± 2.3	19.74 ± 1.8	5	nd	nd	
13575	45.33 ± 3.6*	18.04 ± 1.8*	9	47.44 ± 3**	17.3 ± 1.9**	5	46.41 ± 1.6**	17.55 ± 0.7**	5	45.53 ± 1.2*	17.17 ± 0.7*	5	47.06 ± 2.6	18.79 ± 1.5	3
14015	nd	nd	0	44.92 ± 1.7*	18.82 ± 0.8**	5	41.58 ± 2.3*	20.09 ± 0.8*	5	41.42 ± 2.3	18.16 ± 1.2	5	nd	nd	
15130	49.07 ± 0	17.35 ± 0	2	45.25 ± 1.1**	18.32 ± 0.6**	8	46.75 ± 0.4**	17.8 ± 0.8*	5	43.59 ± 1.9	17.47 ± 0.8*	5	44.87 ± 0.7	20.25 ± 0.6	3
15158	49.78 ± 0.8**	15.45 ± 0.8**	7	47.09 ± 1.6**	17.63 ± 0.9**	8	49.18 ± 1.3**	14.88 ± 1.1**	5	46.09 ± 1**	15.67 ± 0.8**	3	46.67 ± 1.4*	18.38 ± 1.2*	6
15201	nd	nd	0	44.87 ± 1.5*	17.78 ± 1.1**	5	44.93 ± 2.5	14.26 ± 1.8**	5	44.56 ± 2.7*	14.33 ± 1.9**	5	46.96 ± 1.8*	18.6 ± 1.2*	3
15225	nd	nd	0	47.1 ± 2.6**	17.42 ± 1.5**	5	47.62 ± 1.3**	15.97 ± 0.4**	5	44.81 ± 1.6*	15.74 ± 1.1**	5	47.59 ± 2*	17.61 ± 1.1*	3
15251	41.86 ± 2.3	19.36 ± 0.9*	5	46.94 ± 2.6**	17.67 ± 1.4**	8	44.37 ± 0.4	18.53 ± 0.6	5	46.23 ± 1.7*	16.4 ± 1**	5	41.65 ± 1.1	22.08 ± 0.5	3
15310	nd	nd	0	48.1 ± 2.4**	17.26 ± 1.2**	13	45.06 ± 1.2*	18.93 ± 0.7	8	47.69 ± 0.9**	13.26 ± 0.4**	5	42.93 ± 1	20.97 ± 1.1	3
15327	nd	nd	0	47.47 ± 1.1**	17.67 ± 1.1**	5	47.86 ± 1.4**	16.98 ± 0.6**	8	43.31 ± 1.5	16.82 ± 1*	5	nd	nd	
15436	nd	nd	0	47.12 ± 1.2**	18.53 ± 0.3**	5	46.82 ± 1.3**	17.87 ± 0.6*	5	45.02 ± 1.4*	17.03 ± 0.6*	5	51.25 ± 0.6**	16.58 ± 0.5**	3
15439	nd	nd	0	37.37 ± 2.1**	22.05 ± 1.5	5	41.22 ± 1.4*	20.7 ± 0.8*	5	39.51 ± 2.6	19.65 ± 1	5	37.94 ± 2.5	23.12 ± 0.9*	3
15477	51.55 ± 1.4**	17.81 ± 0.3**	3	47.15 ± 2.5**	17.83 ± 1.6**	6	45.75 ± 1.3*	18.2 ± 1.2*	5	43.63 ± 1.2	17.79 ± 0.9	5	nd	nd	
15548	48.88 ± 1.1**	16.14 ± 0.6**	5	44.06 ± 0.8	18.85 ± 0.7*	5	45.84 ± 1.2*	17.71 ± 0.7*	5	44.45 ± 1.3*	16.86 ± 1*	5	43.88 ± 1.9	21.02 ± 0.6	3
15567	45.66 ± 1.8*	16.99 ± 1.7*	5	48.21 ± 1.7**	15.99 ± 1.7**	8	46.51 ± 1.1**	16.67 ± 0.9**	5	46.37 ± 1.9*	15.59 ± 1.1**	5	49.2 ± 1.6*	17.69 ± 2*	3
15906	nd	nd	0	44.12 ± 3	19.17 ± 1.2*	5	44.33 ± 1.5	18.77 ± 0.7	5	46.02 ± 1.1*	16.95 ± 0.3**	5	43.4 ± 3	20.35 ± 1.5	3
15916	nd	nd	0	43.72 ± 7.1	18.29 ± 3.1*	4	44.23 ± 1.7	17.74 ± 1.6*	5	45.11 ± 3*	16.64 ± 1.4*	5	47.74 ± 3.2*	19.17 ± 0.7*	3
15984	48.64 ± 2**	17.58 ± 0.6**	3	47.19 ± 1.1**	17.45 ± 1**	8	46.8 ± 1.3**	17.41 ± 0.6**	5	45.53 ± 2.1*	16.76 ± 0.5**	5	46.87 ± 3.7	20.26 ± 0.7	3
16062	52.1 ± 1**	15.97 ± 1**	5	47.8 ± 2.4*	17.76 ± 1.5**	8	48.51 ± 2.2**	17.85 ± 0.6*	5	45.72 ± 3.9*	16.33 ± 2.6*	5	54.48 ± 0.7**	17.27 ± 0.9*	3
16157	nd	nd	0	44.27 ± 0.9*	19.19 ± 0.6*	5	45.15 ± 1.2*	19.48 ± 0.8	5	45.59 ± 2*	16.04 ± 1.4**	5	43.33 ± 1.5	20.45 ± 0.4	3
16262	nd	nd	0	47.83 ± 2.6**	18.21 ± 1.2**	5	47.41 ± 1.6**	18.23 ± 0.8*	5	41.74 ± 2.7	18.64 ± 0.7	5	nd	nd	
16279	nd	nd	0	45.24 ± 1.4*	10.6 ± 2**	5	45.78 ± 1.7*	7.43 ± 0.7**	5	44.27 ± 2.5	8.41 ± 1.6**	5	38.94 ± 1.3	17.81 ± 3.4	3
16475	nd	nd	0	45.95 ± 1.5**	17.58 ± 1.1**	8	44.79 ± 2.5	17.71 ± 1.6*	8	46.79 ± 1.4**	15.4 ± 1.5**	5	47.57 ± 0.5*	18.96 ± 1.2*	3
16478	nd	nd	0	47.08 ± 0.9**	19.12 ± 1.8	3	47.72	18.21	1	47.16 ± 2.5*	16.33 ± 1.6*	5	49.62 ± 3.5*	18.91 ± 3.2	3
16480	nd	nd	0	48.21 ± 0.8**	15.69 ± 0.3**	5	46.3 ± 2.1*	16.64 ± 0.5**	5	45.35 ± 1*	15.16 ± 0.4**	5	48.15 ± 2.2*	16.97 ± 0.6**	3
16879	47.08 ± 1.3**	16.66 ± 1.1**	5	47.44 ± 0.4*	17.07 ± 1.1*	2	47.95 ± 1.9**	14.66 ± 1.1**	5	44.9 ± 1.7*	15.43 ± 1**	4	49.15 ± 2.7*	16.56 ± 1.2*	3
16921	49.11 ± 1.4**	15.01 ± 1.4**	2	45.89 ± 7.7	18.86 ± 3.9	7	50.43	13.66	2	48.68 ± 1.4**	13.61 ± 1.1**	5	54.44 ± 0.5**	18.25 ± 0.9*	3
17238	nd	nd	0	33.72 ± 2.5**	22.44 ± 0.8*	5	31.24 ± 2.4**	23.78 ± 1**	9	32.91 ± 1.1**	20.54 ± 0.5*	5	35.5 ± 2.9*	22.21 ± 0.4	3
18663	nd	nd	0	49.46 ± 0.6**	15.13 ± 0.5**	3	46.44 ± 1.5**	16.16 ± 1**	5	45.49 ± 1.8*	14.35 ± 1.3**	5	46.19 ± 0.5*	19.02 ± 0.5*	3
18734	nd	nd	0	48.06 ± 1.4**	16.3 ± 0.5**	5	51.53 ± 2.2**	15.3 ± 0.5**	5	48.9 ± 0.4**	13.39 ± 0.7**	5	48.68 ± 0.6*	16.73 ± 0.8**	3
18828	nd	nd	0	46.08 ± 1.7*	16.56 ± 1.3**	5	44.94 ± 1.9	16.82 ± 0.8**	5	45.61 ± 1.3*	15.11 ± 1.7**	5	49.78 ± 0.6*	17.2 ± 0.2**	3
18940	nd	nd	0	46.82 ± 1.1**	14.61 ± 1.3**	5	42.19 ± 2	19.45 ± 0.8	5	42.09 ± 1.4	18.77 ± 1	5	nd	nd	
18974	nd	nd	0	35.76 ± 2.7**	23.21 ± 1*	5	35.67 ± 2.7**	22.54 ± 0.8**	5	36.35 ± 2*	20.6 ± 1.7*	5	39.46 ± 4.4	22.57 ± 2.2	3
21401	45.87 ± 0.5**	18.16 ± 0.8*	5	48.45 ± 2.7**	17.92 ± 1**	8	50.85 ± 1.1**	16.53 ± 0.7**	5	46.38 ± 1.3*	15.76 ± 0.8**	5	51.54 ± 1.6**	16.94 ± 0.9*	3
21406	nd	nd	0	48.32 ± 1.6**	16.24 ± 1.3**	8	47.48 ± 1.2**	17.34 ± 1**	5	48.17 ± 1.9**	14.46 ± 0.4**	4	46.74 ± 1.8*	17.17 ± 1.1*	3
21424	nd	nd	0	43.35 ± 1.9	18.57 ± 0.9**	8	42.98 ± 1.8	19.66 ± 0.9	5	46.33 ± 1.6*	15.17 ± 1.1**	5	42.26 ± 0.2	20.25 ± 0.8	3

21430	nd	nd	0	44.95 ± 2.1*	20.01 ± 0.7	8	46.56 ± 0.6**	18.95 ± 0.8	5	45.53 ± 2.6*	16.39 ± 1.5*	5	46.21 ± 2.5	19.73 ± 1.8	3
21436	nd	nd	0	45.07 ± 1.7*	17.76 ± 0.4**	8	47.61 ± 1**	17.48 ± 0.5**	5	47.01 ± 0.9**	14.62 ± 0.6**	5	44.57 ± 1.9	18.4 ± 1.2*	3
21471	45.59 ± 0.8**	19.29 ± 0.5*	4	42.37 ± 2.6	18 ± 1.2**	8	43.47 ± 2	18.98 ± 0.5	5	nd	nd		nd	nd	
21474	44.65 ± 2.3*	18.73 ± 0.7*	5	46.41 ± 1.8**	16.92 ± 0.6**	8	48.75 ± 3.1**	15.87 ± 1.5**	5	44.8 ± 1.8*	16.58 ± 0.6**	5	47.65 ± 4.6	17.5 ± 1.8*	3
21496	nd	nd	0	45.61 ± 1.5**	17.76 ± 1**	8	46.38 ± 1.9*	16.67 ± 1.2**	5	45.16 ± 0.9*	16.93 ± 0.5**	5	45.04 ± 0.9	19.9 ± 0.7	3
21498	nd	nd	0	44.54 ± 1*	18.69 ± 0.9**	8	45.63 ± 1.4*	17.63 ± 1*	5	44.49 ± 1.2*	16.75 ± 1*	5	46.73 ± 3.7	18.73 ± 0.5*	3
21502	nd	nd	0	46.67 ± 1.7**	17.38 ± 7.6**	5	44.79 ± 1.9	18.67 ± 0.7	5	45.15 ± 1.2*	16.66 ± 0.7**	5	44.91 ± 1.8	18.87 ± 1	3
21503	nd	nd	0	45.63 ± 1.7**	18.39 ± 0.9**	8	46.65 ± 0.7**	17.94 ± 0.3*	5	45.48 ± 1.7*	16.89 ± 0.8*	5	45.52 ± 2*	18.51 ± 0.8*	3
21509	nd	nd	0	44.3 ± 2.4	18.96 ± 1.3*	8	40.43 ± 3.2*	20.21 ± 1.3*	5	39.51 ± 1.4	18.75 ± 1	5	nd	nd	
21527	nd	nd	0	45.76 ± 2.3*	21.3 ± 3	3	47.69 ± 1.4**	17.56 ± 0.9**	5	48.49 ± 1.7**	15.89 ± 1.2**	5	50.82 ± 0.9**	17.97 ± 0.8*	3
21541	nd	nd	0	44.95 ± 3.4	20.66 ± 1.8	8	43.46 ± 3.7*	18.32 ± 1.5**	3	43.47 ± 1.5	16.08 ± 1.3*	5	nd	nd	
21562	nd	nd	0	42.32 ± 3.4	19.33 ± 1.5*	8	45.52 ± 1.9*	17.88 ± 1.6**	5	45.96 ± 3.1*	14.39 ± 0.9**	5	47.84	16.90	2
21572	nd	nd	0	44.35 ± 1.4*	20.54 ± 0.7	7	43.6 ± 1.9	18.61 ± 2.5	5	nd	nd		41.72 ± 2.5	20.96 ± 1	3
21593	nd	nd	0	46.99 ± 2**	17.99 ± 1.1**	6	46.5 ± 1.9**	17.43 ± 0.5**	5	48.02 ± 1.6**	14.65 ± 0.8**	5	43.8 ± 3.6	19.88 ± 1.2	3
21595	nd	nd	0	50.23 ± 1.4**	17.45 ± 1.3**	5	47.39 ± 2.6**	17.91 ± 1.6*	5	47 ± 1.9**	15.96 ± 2*	5	48.87 ± 2.2*	18.27 ± 1.2*	3
21612	49.53 ± 2.7**	15.73 ± 2.2*	5	50.19 ± 1.1**	16.9 ± 1**	8	51.31 ± 1.3**	15.3 ± 1.2**	5	45.40	13.23	2	47.56 ± 1.9*	19 ± 1.5	3
21637	nd	nd	0	46.44 ± 1.3*	16.45 ± 0.9**	2	45.6 ± 1.3*	16.75 ± 1.3**	5	44.61 ± 1.7*	13.89 ± 0.7**	5	47.92 ± 1.1*	18.55 ± 1.2*	3
21683	48.4 ± 1.5**	13.39 ± 1.5**	5	47.28 ± 1.5**	12.2 ± 1.3**	8	49.39 ± 2.6**	9.75 ± 0.6**	5	46.58 ± 1.3**	9.13 ± 5**	5	45 ± 1.6	12.4 ± 0.3**	3
21691	nd	nd	0	43.77 ± 1.5	19.37 ± 0.9**	6	47.39 ± 2.7**	17.92 ± 2.1*	5	46.86 ± 1.9**	15.4 ± 1.8**	5	48.53 ± 1.5*	17.87 ± 1*	3
21693	nd	nd	0	44.95 ± 2.5*	19.86 ± 1.2	8	44.11 ± 0.8	18.99 ± 0.2	5	41.19 ± 2.1	18.07 ± 0.8	5	nd	nd	
21697	nd	nd	0	45 ± 0.7*	18.55 ± 0.6**	5	45.98 ± 1.7*	17.52 ± 1.4*	5	44.54 ± 0.7*	16.55 ± 0.7**	5	43.89 ± 1.6	18.72 ± 0.7*	3
21715	40.75 ± 1.5	17.83 ± 0.7*	2	39.34 ± 1.6*	15.73 ± 4.2*	6	43.61	6.22	2	42.02	3.55	1	42.01 ± 1.9	13.66 ± 3.3*	3
21729	nd	nd	0	47.15 ± 2**	17.97 ± 0.7**	8	45.24 ± 2.6*	18.72 ± 1.2	5	45.61 ± 1*	16.17 ± 0.9**	5	46.82 ± 3.7	18.11 ± 2.1*	3
21768	nd	nd	0	45.99 ± 2.1*	19.52 ± 1.2*	6	46.58 ± 1**	18.07 ± 0.8*	5	50.93 ± 1.2**	13.58 ± 0.9**	5	51.56 ± 1.4**	16.83 ± 1.6*	3
21775	nd	nd	0	47.33 ± 2**	17.79 ± 1**	7	46.31 ± 1.3**	17.25 ± 0.4**	5	45.57 ± 2.4*	15.51 ± 1.3**	5	49.53 ± 1.7*	15.55 ± 1.6**	3
21780	nd	nd	0	46.82 ± 1.4**	17.53 ± 1.4**	8	47.43 ± 1.4**	17.48 ± 0.5**	5	43.81 ± 1.8	16.95 ± 0.7*	5	43.78 ± 3.1	19 ± 0.2*	3
21792	nd	nd	0	45.58 ± 4.2	18.6 ± 2.1*	5	45.13 ± 2.3*	18.13 ± 1.2*	5	44.13 ± 0.5*	15.61 ± 0.6**	4	47.39 ± 1.9*	17.64 ± 1.1*	3
21806	42.16 ± 0.8*	19.38 ± 0.6*	5	46.43 ± 2.5**	17.54 ± 1.4**	8	45.31 ± 1.9*	17.89 ± 0.6*	5	42.62 ± 0.4	17.56 ± 0.6*	5	nd	nd	
21812	nd	nd	0	43.77 ± 1.5	19.09 ± 1.1*	8	44.3 ± 1.8	18.21 ± 0.7*	5	43.77 ± 2.9	17.16 ± 1.3*	5	43.68 ± 1.1	19.17 ± 0.7*	3
21831	50.33 ± 1.2**	16.28 ± 0.7**	5	50.14 ± 1.1**	15.69 ± 0.7**	8	49.82 ± 1.3**	15.64 ± 1.5**	5	47.45 ± 2.6**	14.99 ± 0.9**	5	48.58 ± 3*	17.12 ± 0.5**	3
21855	35.17 ± 1.8*	23.78 ± 1.6*	5	36.95 ± 1.8**	23.35 ± 1.2**	8	38.11 ± 4.2**	22.79 ± 1.7**	5	36.88 ± 2.8*	20.68 ± 1.6*	5	41.68 ± 2	22.07 ± 0.9	3
21887	nd	nd	0	48.76 ± 2.1**	17.23 ± 0.8**	8	49.4 ± 2.6**	15.81 ± 1.8**	5	49.71 ± 0.5**	14.96 ± 0.7**	5	51.07 ± 0.2**	20.42 ± 2.3	3
21888	49.58 ± 1.6**	16.95 ± 0.8**	5	42.15 ± 2.7	20.28 ± 1	8	44.73 ± 0.9	17.9 ± 1*	5	42.64 ± 1.8	18.65 ± 1.4	5	nd	nd	
21934	nd	nd	0	45.27 ± 1.9*	18.63 ± 1.5*	4	46.75 ± 2.1**	17.59 ± 0.6**	5	42.06 ± 1.1	17.35 ± 1.2*	5	nd	nd	
21935	nd	nd	0	43.28 ± 2.7	18.91 ± 2.2*	8	40.52 ± 1.5**	20.37 ± 0.5*	5	42.05 ± 2.3	17.5 ± 1.7	5	nd	nd	
21971	nd	nd	0	48.53 ± 1.7**	16.66 ± 0.8**	8	50.13 ± 1.9**	15.23 ± 0.7**	5	49.56 ± 1.8**	13.95 ± 1.1**	5	44.17 ± 2.4	19.25 ± 0.9*	3
21974	nd	nd	0	48.14 ± 2.1**	18.54 ± 2*	8	50.39 ± 2.6**	15.95 ± 1.4**	5	48.93 ± 0.8**	14.81 ± 0.7**	5	52.41 ± 6.6*	18.17 ± 1.2*	3
21987	nd	nd	0	43.6 ± 2.5	19.42 ± 1.4*	8	43.35 ± 1.8	18.57 ± 1.1	5	43.73 ± 1.7	17.07 ± 0.9*	5	nd	nd	
22008	nd	nd	0	46.6 ± 1.9**	17.47 ± 1.6*	5	47.22 ± 1.1**	15.69 ± 0.8**	5	46.53 ± 2.4*	15.47 ± 1.5**	5	44.03 ± 1.9	19.57 ± 2.1	3
22022	43.28 ± 2.6	16.5 ± 1.4*	3	43.41 ± 1.7	17.65 ± 0.8**	8	43.39 ± 1.4	16.3 ± 0.6**	5	nd	nd		nd	nd	
22073	43.88 ± 3.5**	17.94 ± 3.2	2	46.3 ± 0.8*	16.73 ± 1.5**	2	44.20	16.55	1	nd	nd		nd	nd	
22080	38.19 ± 1.4*	21.91 ± 1	5	41.83 ± 3.2	20.27 ± 2.3	7	40.77 ± 1.2**	19.89 ± 0.7	5	nd	nd		nd	nd	
22081	51.15 ± 2.3**	15.01 ± 2.1*	5	51.38 ± 2.7**	14.72 ± 1.4**	8	53.3 ± 4.2**	12.46 ± 2.7**	5	52.17 ± 1.3**	11.67 ± 0.7**	5	57.24 ± 5.6*	14.98 ± 0.3**	3
22083	44.1 ± 2.7*	18.23 ± 1*	5	48.43 ± 3.1**	16.89 ± 1.1**	8	46.48 ± 1.2**	16.96 ± 0.5**	5	45.13 ± 2.4*	15.23 ± 1.9**	5	45.57 ± 1.5*	18.3 ± 1*	3
22094	nd	nd	0	44.64 ± 2.3*	18.17 ± 2.2*	5	47.31 ± 1.7**	15.18 ± 1.6**	5	46.27 ± 2*	11.58 ± 2**	5	47.72 ± 2.8*	19.14 ± 1*	3
22102	nd	nd	0	50.14 ± 0.9**	13.74 ± 1**	7	47.77 ± 1.1**	12.84 ± 2**	5	46.97 ± 1.9**	12.66 ± 1.4**	5	49.77 ± 5.6*	16.85 ± 3*	3
22143	nd	nd	0	44.89 ± 2.7*	19.09 ± 0.7*	8	45.05 ± 2.1	18.76 ± 1	5	45.79 ± 1.6*	17.44 ± 0.7*	5	45.47 ± 2.5	18.94 ± 1.2*	3
22166	45.76 ± 2.8*	16.79 ± 1.8*	5	46.85 ± 2.5**	17.16 ± 1.6**	8	46.74 ± 0.8**	16.76 ± 0.6**	5	42.91 ± 2.3	16.19 ± 1.2**	5	47.95 ± 1.8*	17.29 ± 1.1*	3
22174	46.15 ± 2.4*	18.62 ± 1.5*	5	46.53 ± 2.2**	17.24 ± 1.9**	8	48.44 ± 0.9**	16.37 ± 0.8**	5	46.13 ± 2.3*	16.98 ± 1.9*	5	46.58 ± 2.1*	16.68 ± 0.8**	3
22187	51.99 ± 1.7**	15.88 ± 0.9*	5	52.8 ± 1.4**	15.31 ± 1**	8	51.69	15.99	2	50.14 ± 1.5**	14.37 ± 1.5**	5	53.22 ± 0.4**	16.5 ± 1.2*	3

22189	45.4 ± 2.7*	17.88 ± 1.3*	5	51.18 ± 1.7**	14.92 ± 0.6**	8	52.13 ± 1.5**	14.22 ± 0.7**	5	44.94 ± 2.2*	16.69 ± 1.1*	5	43.67 ± 2.5	19.11 ± 1*	3
22226	41.69 ± 1.3	14.88 ± 0.8**	5	43.71 ± 1.2	16.04 ± 1.3**	8	42.36 ± 1.4	15.5 ± 0.8**	5	nd	nd		nd	nd	
22291	47.16 ± 3*	16.35 ± 1.4**	5	48.38 ± 3.8*	16.71 ± 2**	5	45.6 ± 1.9*	17.31 ± 1.9*	5	44.47 ± 3.2	15.94 ± 1.5**	5	48.28 ± 2.7*	18.36 ± 1.3*	3
22332	48.54 ± 1.2*	15.8 ± 1.2**	2	43.12 ± 1	19.01 ± 0.9*	3	42.87 ± 0.5	19.81 ± 0.9	5	41.3 ± 2.6	17.37 ± 1.3*	5	nd	nd	
22351	44.09 ± 1.3*	19.92 ± 1.1	5	44.85 ± 2.9*	18.72 ± 1.2*	8	43.89 ± 1.7	19.21 ± 0.9	5	nd	nd		nd	nd	
22426	47.06 ± 1.6**	18.48 ± 1.1*	5	47.4 ± 3.7*	18.01 ± 2.8*	5	46.86 ± 3.1**	18.16 ± 1.3*	5	45.6 ± 2.5*	17.25 ± 0.9*	4	44.5 ± 1.5	20.62 ± 0.9	3
22492	46.27 ± 2.8*	15.4 ± 1.4**	5	43.38 ± 1.7	17.44 ± 1.5**	8	43.67 ± 1.5	15.36 ± 0.4**	5	45.86 ± 1.1*	12.56 ± 1.1**	5	43.18 ± 1.4	17.24 ± 4	3
22510	46.37 ± 1.9**	16.46 ± 1.4**	5	43.53 ± 1.4	18.02 ± 0.8**	7	43.08 ± 1.2	17.28 ± 1**	5	39.29 ± 2.9	16.2 ± 1.6*	5	nd	nd	
22705	nd	nd	0	43.35 ± 3.8	19 ± 2.2*	8	44.38 ± 1	17.62 ± 1*	5	42.7 ± 0.8	15.58 ± 1.2**	5	nd	nd	
22811	48.92 ± 2.7**	15.47 ± 1.5**	5	46.75 ± 2.3*	17.38 ± 2.5*	2	43.43 ± 1.1	18.07 ± 0.2*	5	nd	nd		nd	nd	
22852	45.12 ± 3.5*	17.45 ± 1.4*	5	49.46 ± 2.3**	15.92 ± 1.1**	8	40.44 ± 4.7**	18.58 ± 2.1	5	nd	nd		nd	nd	
22888	44.96 ± 0.2*	17.76 ± 0.2*	2	43.53 ± 2.2	19.42 ± 0.8*	8	44.55 ± 0.8	19.26 ± 0.5	5	48.21	14.82	2	45.31 ± 2.5*	20.39 ± 1.6	3
CL0J	nd	nd	0	43.05 ± 1.3	19.33 ± 1.2	5	41.42 ± 1.4*	19.1 ± 0.5	8	41.36 ± 1.5	18.17 ± 0.8	5	41.12 ± 1	20.29 ± 0.6	6
DBK	52.3 ± 0.9**	15.05 ± 0.3**	4	52.91 ± 1.4**	14.48 ± 1.1**	16	52.26 ± 1.1**	14.16 ± 1**	5	50.41 ± 1**	13.25 ± 0.7**	4	54.81 ± 2.2**	12.37 ± 1.5**	7
Kinb.	48.37 ± 1**	17.02 ± 0.7**	4	50.62 ± 1.7**	15.21 ± 1.2**	8	49.32 ± 0.9**	14.59 ± 0.6**	6	49.93 ± 1.2**	13.81 ± 0.9**	5	49.69 ± 2.7**	16.7 ± 1.3	10
LG04	nd	nd	0	42.21 ± 1.6**	18.92 ± 0.8	10	40.9 ± 1.2**	18.38 ± 0.7**	16	39.31 ± 2.4	17.71 ± 1.1*	5	41.13 ± 1.7	18.93 ± 0.6**	6
W82	40.32 ± 0.9	20.66 ± 0.5	4	42.36 ± 1.7	20.8 ± 1.1	10	42.85 ± 1.2	19.49 ± 1.2	15	41.61 ± 2.1	18.92 ± 0.9	8	41.97 ± 2.4	21.03 ± 1*	5

nd – not determined

Average and standard deviation for protein and oil levels (dry weight basis) for bulk seed samples from individual plants of mutant lines over 5 seasons. ** - significantly different from the control (by two-tailed type-2 *t*-test at $p < 0.001$, * significantly different from the control at $p < 0.05$. Red color indicates mutant was replaced by backcross progeny after backcrossing.
